# Patient's Payments

**Published:** 1920-06-19

**Authors:** John Hy. Johnson

**Affiliations:** (Captain), *Secretary.* Royal Westminster Ophthalmic Hospital, King William Street, West Strand, W.C. 2.


### THE EDITOR'S LETTER-BOX.

**Correspondence on all subjects is invited, but we cannot in any way be responsible for the opinions expressed by our correspondents, who must give their name and address as a guarantee of good faith, but not necessarily for publication. Correspondents are reminded that brevity of style and conciseness of statement greatly facilitate early insertion.**

### Patient's Payments.

*To the Editor of* The Hospital.

Sir,—It will doubtless be of interest to you to be informed that the Committee of Management of this hospital—which has treated patients for upwards of 100 years, entirely free and without "letters of recommendation"—has given very careful consideration to the question of "patients' payments," and at a recent meeting it was decided that all patients should pay for their medicines, as from July 1 next, on the basis of 6d. per week, with a maximum of 1s. 6d. if more than three weeks' medicine is supplied.

With regard to in-patients paying something towards their maintenance, it has not yet been decided, and the question left in abeyance, at any rate for the present.

The Committee feel that the classes availing themselves of hospital treatment have improved their financial condition so considerably since the war that a payment as stated above will be no hardship.—I am, Sir, yours faithfully,

John Hy. Johnson,

(Captain), *Secretary.*

Royal Westminster Ophthalmic Hospital,
King William Street, West Strand, W.C. 2.
June 15, 1920.

